# Ontology Alignment Architecture for Semantic Sensor Web Integration

**DOI:** 10.3390/s130912581

**Published:** 2013-09-18

**Authors:** Susel Fernandez, Ivan Marsa-Maestre, Juan R. Velasco, Bernardo Alarcos

**Affiliations:** Department of Computing Engineering, University of Alcala, Superior Polytechnic School, University Campus, Alcalá de Henares 28805, Madrid, Spain; E-Mails: ivan.marsa@uah.es (I.M.-M.); juanramon.velasco@uah.es (J.R.V.); bernardo.alarcos@uah.es (B.A.)

**Keywords:** semantic sensor web, ontology alignment, fuzzy logic

## Abstract

Sensor networks are a concept that has become very popular in data acquisition and processing for multiple applications in different fields such as industrial, medicine, home automation, environmental detection, *etc.* Today, with the proliferation of small communication devices with sensors that collect environmental data, semantic Web technologies are becoming closely related with sensor networks. The linking of elements from Semantic Web technologies with sensor networks has been called Semantic Sensor Web and has among its main features the use of ontologies. One of the key challenges of using ontologies in sensor networks is to provide mechanisms to integrate and exchange knowledge from heterogeneous sources (that is, dealing with semantic heterogeneity). Ontology alignment is the process of bringing ontologies into mutual agreement by the automatic discovery of mappings between related concepts. This paper presents a system for ontology alignment in the Semantic Sensor Web which uses fuzzy logic techniques to combine similarity measures between entities of different ontologies. The proposed approach focuses on two key elements: the terminological similarity, which takes into account the linguistic and semantic information of the context of the entity's names, and the structural similarity, based on both the internal and relational structure of the concepts. This work has been validated using sensor network ontologies and the *Ontology Alignment Evaluation Initiative* (*OAEI*) tests. The results show that the proposed techniques outperform previous approaches in terms of *precision* and *recall*.

## 1. Introduction

The increasing miniaturization of computers raises the idea of developing extremely small, inexpensive computers that communicate wirelessly and are organized independently. A sensor network is a network of tiny computers (nodes) equipped with sensors which collaborate on a common task [1]. These nodes have certain sensory capabilities and wireless communications that enable *ad hoc* networking without any preset physical infrastructure or central administration. Sensor networks are a relatively new concept in data acquisition and processing for multiple applications in different fields such as industry, medicine, home automation, military environments, environmental detection, *etc.* Their main features (to be small, cheap, autonomous, easy to deploy, self-configurable and able to perform efficient energy management) have made sensor networks a very active research field, in which systems as diverse as Berkeley Motes [2], Pico-Radio [3], Smart-Dust [4] and WINS [5] have been developed.

With the proliferation of small communication devices with sensors that collect environmental data, semantic Web technologies are becoming closely related to sensor networks. O'Reilly even states that Web 3.0 is the network of sensors instead of the Semantic Web [6]. The linking of elements from Semantic Web technologies with sensor networks dates back to 2005 and was called “Semantic sensor nets” [7]. In 2008 a framework named Semantic Sensor Web [8] was proposed. One of the main features of this framework is the use of ontologies.

An ontology for sensor networks should capture information about sensor capabilities, performance, and the conditions in which it can be used, allowing the discovery of data for different purposes and in different contexts. Several ontologies have been developed to define the capabilities of the sensors and sensor networks. Amongst them we can mention CSIRO Sensor Ontology [9], OntoSensor [10], SWAMO [11], MMI Device Ontology [12], SensorML Processes [13], CESN [14], WISNO [15], A3ME [16] and Ontonym-Sensor [17].

One of the main challenges of using ontologies in such networks is related to the semantic heterogeneity. The key idea here is to provide mechanisms to integrate and exchange knowledge from heterogeneous sources. More precisely, what we want is to provide techniques to enable the processing, interpretation and sharing of data from sensor networks which use different data models, or whose information is organized into different ontological schemes. There are many application scenarios where solving the problem of semantic heterogeneity may have a big impact. The exchange of information and services in the area of the Internet of Things may be one of these domains, since in many cases there is a vast variety of devices which need to be monitored through software entities such as multi-agent systems. Another application scenario for this kind of techniques is scene of forest fire control, due to the need for early detection to reduce the impact these fires have on ecosystems. Currently there are systems in forest fire detection using sensor networks [18] that measure different factors such as temperature, humidity, smoke concentration in air, *etc.* In these cases, it may be useful to be able to interconnect the sensors deployed in the forests with the ones carried by the emergency response teams either in trucks or personal units (e.g., GPS). The prompt, efficient, and seamless information exchange between these sensors would allow for a better distribution of resources to facilitate an adequate control of the fires.

From the discussion above it is clear that semantic sensor networks interoperability is a crucial challenge in a number of real world domains. This challenge can be addressed by finding relationships between entities belonging to different sensors ontologies and taking advantage of these relations. This is closely related to the problem of ontology alignment, which can be defined in one sentence as follows: given two ontologies that describe a set of discrete entities, ontology alignment is the process of finding correspondences between them [19]. One of the key factors to consider for this process is to find similarity measures that allow one to obtain precise correspondences between entities of different ontologies. For this reason, research in this area has focused primarily on similarity metrics.

Our main objective in the field of sensor networks is to align the largest possible set of ontologies and implement a tool similar to WordNet [20] to automatically allow accessed to these alignments. This tool will enable any application requiring interaction between different ontologies to directly accessed the repository and get all kinds of relations between different entities quickly, without performing the mapping (which can be computationally expensive). This would greatly facilitate semantic interoperability in this domain.

As a first step in this line, this work presents a multilayer Fuzzy Rule-Based System for ontology alignment applied to Semantic Sensor Networks. The method described combines two similarity measures for establishing correspondences between entities from different sensor ontologies. The first measure is the terminological similarity, which takes into account the linguistic and semantic information in the context of the concept names, and the second is the structural similarity, which uses both the taxonomic hierarchy of concepts and their internal structure, represented by their properties, types and cardinality restrictions. The main factor which distinguishes this work from the general algorithms of ontology alignment lies in the method of calculating the semantic similarity. We carry out a search of the concepts contextualized within the sensor subdomain of *DBPedia* through automatic search queries.

The rest of the paper is organized as follows: Section 2 provides a brief introduction to ontologies and presents an overview of sensor ontologies. In Section 3 we discuss the most relevant related works in ontology alignment. Section 4 describes the fuzzy rule-based system for the ontology alignment and the proposed similarity measures. Finally, Section 5 is dedicated to the experimental evaluation results. The last section summarizes the conclusions and suggests future lines of research.

## 2. Ontologies in Sensor Networks

Using logical processing tools in the environment of sensor networks allows answering queries, to infer information, to search and to identify resources, among other benefits. To achieve this, reasoning and inference in the analysis of specifications and links between entities and data are required. This enables users to develop and adapt sensor networks, abstracting from low-level details of network and multiple devices. One of the most suitable logical processing tools for this environment is the use of ontologies.

An ontology provides a common vocabulary of a domain of knowledge and defines the meaning of the terms and the relations between terms in different levels of formality [21]. The components of ontologies are classes (concepts), relations, axioms and individuals. The classes or concepts in the ontology represent any entity that provides some information and contains properties. Relations represent interactions between classes, such as inheritance (usually called taxonomic relation), and individuals are concrete instances of a particular class.

In the field of sensor networks, there are several ontologies to organize the concepts and relations of the domain, and a number standards and rules have been developed for this purpose. The Open Geospatial Consortium (OGC) [22] has developed Sensor Web Enablement (SWE) [23] as a suite of standards to enable developers to make all types of networked sensors, transducers and sensor data repositories discoverable, Accessedible and usable via the Web or other networks. The first SWE standard was the Sensor Model Language (SensorML) [13], which provided standard models for describing processes within sensor and observation processing systems.

Based on SensorML and other standards, the OntoSensor [10] ontology emerged. It was intended as a general knowledge base of sensors for query and inference. OntoSensor covers a wide range of concepts, class definitions and individuals and can describe the platform a sensor is attached to, but can only describe generic *part-of* relations. This ontology provides high expressiveness for data and the ability to organise sensors into a hierarchy of sensing concepts.

The Commonwealth Scientific and Industrial Research Organisation (CSIRO) has developed a generic sensor ontology [9] for describing sensors and deployments. It is intended to be used in data integration, search, classification and workflows. The CSIRO ontology, like OntoSensor, covers a wider range of concepts than the other ontologies. Both are able to describe most of the spectrum of sensor-related concepts. However the CSIRO ontology can describe more complex *part-of* relations such as composition and structure, CSIRO ontology can describe sophisticated forms of structural and sequencing composition, with, for example, sequence, conditional and repetition for process composition.

Some of the existing ontologies have been designed for specific application industries but their use has spread to many other areas, becoming generic ontologies. The Marine Metadata Interoperability (MMI) Device [12] is an ontology of oceanographic devices, sensors and samplers. It focuses on systems, the components of a system and how those components are organised. It can be seen as ontology for describing the structure and process of measurement taking systems, and also includes concepts for describing measurements. MMI is work-in-progress and it's likely that their scope will be extended, adding concepts ranging from physical properties and limits of the sensor to communication information and software. Another ontology, SWAMO [11] was designed to describe agent, process and sensors enabling dynamic and composable interoperability of sensor web products and services. This ontology can describe *part-of* relations for systems and a form of process chaining. MMI and SWAMO extend the analysis along a third dimension, from measurements and sensor types to systems and structure. The Coastal Environmental Sensing Networks (CESN) [14] is an ontology for sensor networks for coastal observing. CESN ontology provides sensor types, a description logic (DL) and a rule-based reasoning engine to make inferences about data and anomalies in measurements. This ontology is almost entirely a description of sensor types.

Another ontology is An Agent-Based Middleware Approach for Mixed Mode Environments (A3ME) [16], which was created as a basic classification for self-description and discovery of devices and their capabilities in heterogeneous networks including resource constrained sensor nodes. This ontology is considered moderately broad with respect to sensors and covers: device classification (tag, mote, mobile, workstation, server, vehicle and multimedia), general capability classes (sensor, actuator, communication, storage, computing, energy) and subclasses for each capability class.

Ontonym is a set of upper ontologies that represent core concepts in pervasive computing (time, location, people, sensing, provenance, events, device, resource). A newest ontology in this group is Ontonym-Sensor [17], which provides high level descriptions of a sensor and its capabilities (frequency, coverage, accuracy and precision pairs) and descriptions of sensor observations (observation-specific information, metadata, sensor, timestamp, time period over which the value is valid, rate of change). This ontology is a basic hierarchy of concepts with some properties that must be extended to characterise any specific sensor and its data.

Recently the Semantic Sensor Networks Incubator Group [24] belonging to the World Wide Web Consortium (W3C) has developed the Semantic Sensor Network (SSN) ontology [25] which can model sensor devices, systems, processes, observations and knowledge of the environment. The SSN ontology has begun to achieve broad adoption and application within the sensors community. The main classes of the SSN ontology have been aligned with classes in the DOLCE Ultra Lite (DUL) [26] foundational ontology to facilitate reuse and interoperability. However the rest of existing ontologies in sensor networks domain do not propose any kind of alignment with any other, making interoperability between them difficult.

## 3. Related Work in Ontology Alignment

There are a number of previous works aimed at ontology alignment in general, which have made interesting contributions. SMART [27], PROMPT [28] and PROMPTDIFF [29] are tools that have been developed using linguistic similarity matches between concepts and specific heuristics to identify further matches. These tools do not take into account the properties or relations of concepts and assume that all ontologies belong to a specific knowledge model, which makes the systems not applicable to other knowledge models.

Other approaches use probabilistic methods, such as CODI [30], which produces mappings between concepts, properties, and individuals. CODI is based on the syntax and semantics of Markov Logic. GLUE [31] employs machine-learning techniques to find mappings. In [32] a probabilistic framework for automatic ontology mapping based on Bayesian Networks is proposed. This approach only takes into account the probability of occurrence of concepts in the Web, which makes it fail if two very similar concepts have not the same level of popularity.

There are more recent works that combine lexical similarity with other techniques. One of them is ASMOV [33], which iteratively computes the similarity by analyzing lexical elements, relational structure, and internal structure. AgrMaker [34] comprises several matching algorithms that can be concept-based or structural. The concept-based matchers support the comparison of strings and the structural matchers include the descendants' similarity inheritance. In AgrMaker the structural similarity depends absolutely on linguistic relationships, which means that correct results are not obtained in ontology concepts which are not linguistically similar, even when the ontologies are very similar in structure.

In Eff2Match [35] the alignment process consists of four stages: *Anchor Generation*, where entities are identified using an exact string matching technique; *Candidates Generation*, where they look for entities using a vector space model approach; *Anchor Expansion*, where more equivalent pairs of entities are identified using terminological methods; and *Iterative Score Boosting*, where more pairs of equivalent concepts are identified using the expanded anchor set. This system yields poor results in ontologies which differ linguistically and structurally, and thus it is difficult to get correct results when aligning heterogeneous ontologies.

GeRMeSMB [36] is the integration of two tools; GeRoMeSuite offers a variety of matchers which can match ontologies and schemas in other modeling languages such as XML or SQL; and SMB mainly works on the similarity matrices produced by GeRoMeSuite. It improves the clarity of the similarity values by reinforcing “good” values and penalizing “bad” values to increase the precision of the result. This is a very generic system and does not work very well on real ontologies in specific domains.

SOBOM [37] deals with ontologies from two different perspectives: hierarchical structures in the ontology and other relationships, combining the results of every step in a sequential way. If the ontologies have regular literals and hierarchical structures, the system can achieve satisfactory alignments and avoid missing alignment in many partitioning matching scenarios. If the literals of the concepts are missed, the system will get bad results.

One of the most complete approaches in ontology matching is S-Match [38], an open source semantic matching framework that provides several matching algorithms. It includes components for transforming tree-like structures into lightweight ontologies, where each node label in the tree is translated into a propositional description logic (DL) formula, which univocally codifies the meaning of the node. Among the matching algorithms that are implemented in S-Match we can mention three: (1) a basic general-purpose semantic matching algorithm; (2) the minimal semantic matching algorithm, which exploits additional knowledge encoded in the structure of the input; and (3) a type of semantic matching which produce a similarity score and a mapping preserving structural properties.

From the discussion above, we can conclude that so far most of the existing systems for ontology matching have focused primarily on calculating similarities between the names of concepts and properties, but there are few studies that exploit the hierarchical structure of classes. Despite the many contributions that have been developed in the context of ontology alignment, none offers a complete matching solution due to the structural complexity of the ontologies. There is no integrated solution that has proven to be robust and effective enough to be taken as a basis for future development [19]. Another front in this field is the improvement of similarity measures in order to obtain more precise values minimizing human intervention in the process.

An additional challenge is managing uncertainty, which still requires more work, despite having been addressed in some approaches, because none has solved the problem effectively and automatically. In the specific case of the similarity calculation in the domain of sensor networks there are concepts which are have not yet been included into a general lexicon and there are no lexical tools defining linguistic relations between all the concepts to facilitate the mappings. Our proposal focuses on providing various similarity measures for ontology mapping using specific information of the context of sensor networks. Due to the imprecise nature of the measures, we decided to use fuzzy logic techniques to combine them to obtain more accurate alignments.

## 4. The Fuzzy Rule-Based System

**Figure 1.**
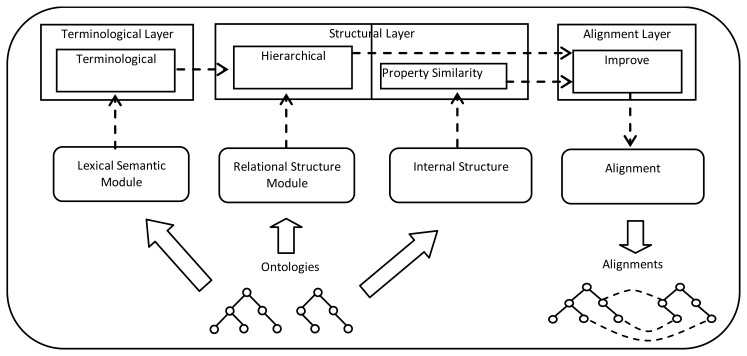
Architecture of the Fuzzy Rule-Based System.

Fuzzy Rule-Based Systems are an extension to classical rule-based systems. They deal with “IF-THEN” rules whose antecedents and consequents are composed of fuzzy logic statements instead of classical logic ones. They have been successfully applied to a wide range of problems in different domains with uncertainty and incomplete knowledge [39]. A Fuzzy Rule-based System consists of four distinct parts: the *knowledge base* that contains the data and the rules of the system; the *inference engine* that is responsible for drawing the conclusions from the symbolic data that have arrived using the rules governing the system in which it works; and the *fuzzification* and *defuzzification* interfaces which have the function of converting real input values into fuzzy values and vice versa. The proposed system is called *FuzzyAlign*. It is a multi-layer fuzzy rule-based system which uses three different layers to perform the ontology alignment process: a terminological layer, a structural layer and an alignment layer. The output values of each layer serves as input to the upper one and each layer provides an improvement in the calculation of the similarity between concepts. Figure 1 shows the architecture of the fuzzy rule-based system.

### 4.1. Terminological Layer

The terminology of the names of concepts and properties in ontologies provides valuable information about the entities that they model, and therefore it is the first item to consider when attempting to find correspondences between them. Generally, in the ontology alignment methods the names are processed as separate elements using string-based techniques [27–29], such as string distance functions. Authors have also employed other techniques combined with the use of external linguistic resources such as thesauri and specialized directories in different domains. But still there are very few studies that use semantic context information to calculate the terminological similarity among ontology entities. We propose a contextual approach to improve the values of similarity between the entities' names taking into account not only their construction from the lexical point of view but also the context in which the terms are used within the ontologies. This approach is described in the following sections.

#### 4.1.1. Linguistic Similarity

Lexical is the strongest indicator of similarity between entities, because usually the ontology developers within the same domain of knowledge use linguistically related terms to express equivalent entities [40]. In this work two types of lexical similarity are computed over the concepts: the first one based on the synonyms, and the other based on the derivationally related forms of the words. The WordNet directory [20] has been used as external lexical tool to get the data needed for the computation of the similarities. WordNet [20] is an electronic thesaurus based on psycho-linguistic theories to define the meaning of words and model associations of meanings with words. WordNet uses three databases, one for nouns, one for verbs and the third for adjectives and adverbs. The basic elements are the synonym sets, called *synsets*. A *synset* denotes a concept or a sense of a group of terms and provides different linguistic relations depending on the part of speech (nouns, verbs, adjectives and adverbs). The proposed algorithm for the linguistic similarity calculation given the concepts *a* and *b* consists of the following steps:

1. *Normalization*: In this work we normalize using *tokenization*, which is the process of converting the strings into tokens or sequences of lexical components; and the removing of meaningless words (*stop words*).
2. *Relation*: In this step the lists of synonyms and words derived from each concept are obtained using WordNet as lexicon.
3. *Stemming*: In this step the *Porter Stemming Algorithm* [41] is applied to obtain the morphological roots of the words from the lists of synonyms and derivative words.
4. *Similarity*: Finally the linguistic similarity of synonymy and derivationally related words between the concepts is calculated according to Equation (1):
  (1) $$S(a,b)=\min[\frac{\left|A\cap B\right|}{\left|A\right|},\frac{\left|A\cap B\right|}{\left|B\right|}]$$
  where *A* and *B* be are the sets of roots of synonyms or words derived from each concept obtained in the third step. The first ratio indicates the fraction of overlap of the set *A* with respect to set *B*, and the second ratio indicates the overlap fraction of the set *B* relative to the set *A*. As both sets are not necessarily having the same number of elements, the minimum value among their degrees of overlap is chosen as an indicator of their similarity.

For example if we need to calculate the derivational linguistic similarity of the concepts *a* = *sensor* and *b* = *sensing device*, the result is show in Table 1.

#### 4.1.2. Semantic Similarity

The semantic similarity is incorporated with the aim of adding context information of the concepts in the mapping process. This measure is calculated using the *Jaccard Coefficient* [42], which is one of the most used binary similarity indexes. Given two sets of data this coefficient is defined as the size of the intersection of the sets divided by the size of the union. For two binary observations *i* and *j*, the *Jaccard Coefficient* is calculated by:

(2) $$S_{\text{Jaccard}}(i,j)=\frac{a}{a+b+c}$$

where *a* is the number of times that both observations have the value 1, *b* is the number of times observation *i* has value 1 and observation *j* has value 0, and *c* is the number of times observation *i* has value 0 and observation *j* has value 1.

To calculate the semantic similarity, subsequent searches of documents from the web are performed, specifically in *DBpedia* [43], using a *DBpedia* API for queries [44]. For the alignment of general-purpose ontologies, the search function of the API is configured so that queries are performed on all the databases of *DBpedia*, without restriction. However, in the case of domain-specific ontologies as sensors, this function is configured to perform contextualized queries.

For the process of contextualization in the domain, the first step is finding a context globally and is simply to focus on the domain in question, in this case the sensors field. To this end, we restrict the search queries only to the *sensor* category of the macro ontology of *DBpedia*, and so we accessed the hierarchies of concepts belonging to this specific domain. This is achieved with a little change in the source configuration parameter of the search function, which indicates the specific domain where we want to make the queries (http://dbpedia.org/ontology/sensor). This guarantees the relevance of the results in the domain of sensors.

The next step is a local contextualization, and as its name suggests, it takes into account the context of each concept in the ontology (*i.e.*, relations with other concepts.) To ensure that the search only returns relevant documents to the local context of the entities, the search query is formed by combining all the terms on the path from the root to the current node in the taxonomy. Let us assume that the set *A*^+^ contains the elements that support entity *A*, and the set *A*^−^ contains the elements that support the negation of *A*. Elements in *A*^+^ are obtained by searching for pages that contain *A* and all *A*'s ancestors in the taxonomy, while elements of *A*^−^ would be those where *A*'s ancestors are present but not *A*. For each pair of entities *A* and *B*, three different counts are made: (a) the size of *A*^+^*∩B*^+^; (b) the size of *A*^+^*∩B*^−^, and (c) the size of *B*^+^*∩A*^−^.

**Figure 2.**
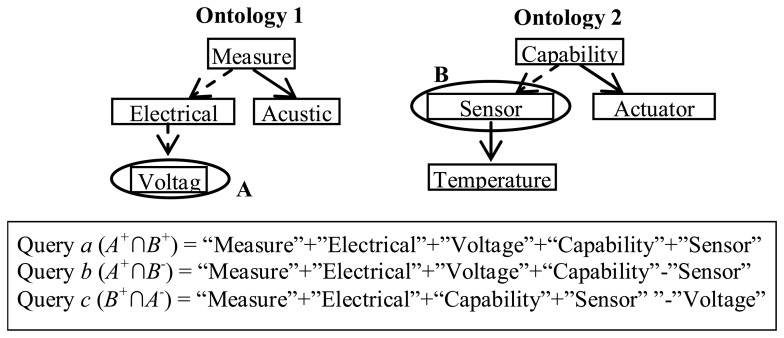
Example search queries for computing semantic similarity between two concepts from different sensor networks ontologies.

Once these values are obtained for each pair of origin and destination ontology entities, their similarity is calculated using Equation (2). Figure 2 shows fragments of two ontologies in the domain of sensors and the search queries that would be formed to calculate the semantic similarity between the concepts *Voltage* and *Sensor*.

#### 4.1.3. Terminological Similarity

**Figure 3.**
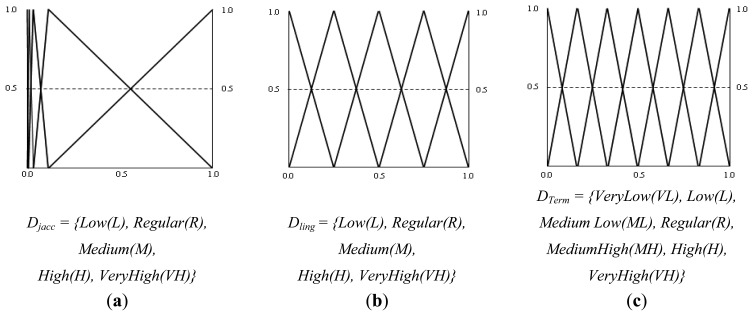
Membership functions and linguistic terms set. (**a**) Semantic similarity; (**b**) Linguistic similarity; (**c**) Terminological similarity.

As outlined above, the terminological layer of the fuzzy rule-based system is responsible for carrying out the process of computing the similarity between the entity names within the ontologies, combining linguistic similarity with the semantic elements of the context of the entities. This layer receives as inputs the values of the linguistic similarity and the semantic similarity computed in the lexical-semantic module. The output variable represents the terminological similarity. To define the triangular membership functions of the fuzzy rule-based system, equally spaced fuzzy sets were used, except in the case of the variable representing the semantic similarity due to the distribution of the values. In this case the quartiles of the data were used to narrow the membership triangles. Figure 3 shows the triangular membership functions and the set of linguistic terms defined for the three variables.

### 4.2. Structural Layer

The structural level matching techniques are the ones which that work consider the relationships between the elements within the ontologies. We propose two different similarity measures from the structural point of view: the first using the relational structure of concepts in the ontology, specifically the taxonomic hierarchy, and the second using the information of the internal structure of concepts, including their properties, types and cardinality restrictions. The structural layer performs two key tasks related to the structure of ontologies. One is the calculation of the similarity between the concepts taking into account the taxonomic hierarchy, and the other is the computation of the similarity between the properties of the concepts independently.

#### 4.2.1. Relational Structure Similarity

**Figure 4.**
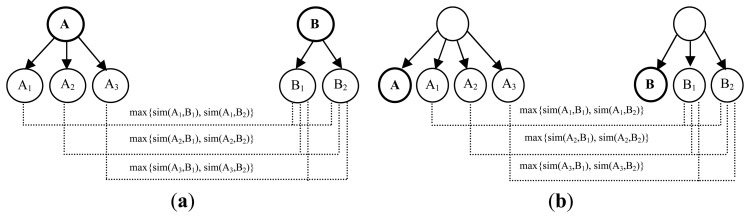

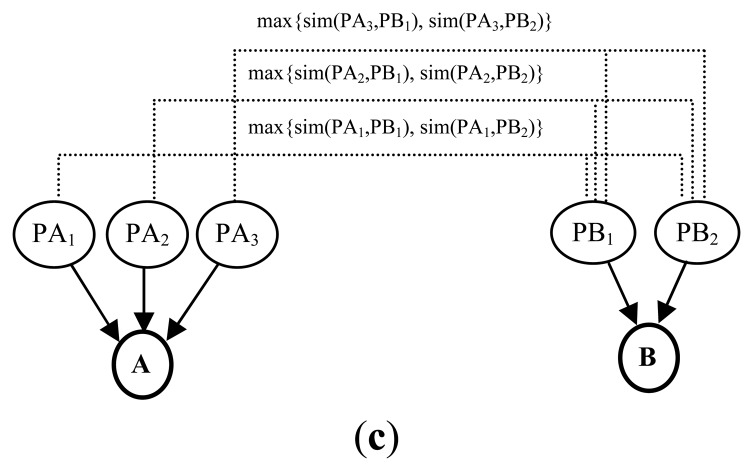
(**a**) Hierarchical Similarity of descendants; (**b**) Hierarchical Similarity of siblings. (**c**) Hierarchical Similarity of parents.

The relational structure similarity is calculated using the taxonomic hierarchy of concepts in the ontologies. Thus, the similarities of the parents, siblings and descendants of the concepts in the taxonomies are considered, in addition to the terminological similarity. The *Hierarchical Similarity* is defined as the influence that the siblings, parents and descendants have on the final similarity of concepts, starting from the idea that if two concepts *A* and *B* are similar, and their siblings, descendants, or parents are also similar, it is likely that *A* and *B* are equivalents. Figure 4 shows how to calculate the three types of *Hierarchical Similarity* (related to siblings, parents and descendants) for concepts *A* and *B*. Given the concepts *A* and *B* in two different ontologies, being *n* the number of parents, descendants or siblings of the concept *A*, and *m* the number of parents, descendants or siblings of the concept *B*, and *A_i_* and *B_j_* being the *ith* and *jth* descendant, parent or sibling of the concepts *A* and *B* respectively, the *Hierarchical Similarity* is defined as:

(3) $$\text{Sim}(A,B)=\frac{1}{n}\overset{n}{\underset{i=1}{\text{∑}}}\max{\left\{\text{Sim}(A_{i},B_{j})\right\}}_{j=1}^{m}$$

The hierarchical similarity system receives as input the terminological similarity calculated in the previous layer of the fuzzy system and the hierarchical similarities that descendants, parents and siblings of the concepts provide, which are calculated in the relational structure module, and the output is the hierarchical similarity of the concepts. For the five variables the same fuzzy sets and membership functions that were shown in Figure 3c were defined.

#### 4.2.2. Internal Structure Similarity

This similarity measure is based on the internal structure of concepts, which is influenced by other factors besides their names and relationships such as the similarity of their properties, and cardinality. Similar to the calculation of the relational structure similarity, this work start from the idea that if two concepts are similar, they have the same number of properties, and these properties have a high degree of similarity, then it is very likely that they are equivalent concepts and we reinforce the value of their similarity. In contrast, if two concepts have some resemblance, but they have not the same numbers of properties or these properties are not similar, their similarity value is decreased. For each pair of concepts (classes) *A* and *B*, the *Property-wise* similarity is defined as the value that the properties bring to the final class similarity. It is calculated in the same way that the *Hierarchical Similarity*, averaging the maxima of the similarities between the properties of the concept *A* and the properties of the concept *B* as shown in Equation (4), where *PA_i_* is the *ith* property of the concept *A*, and *PB_j_* is the *jth* property of the concept *B*.

(4) $$\text{Sim}(A,B)=\frac{1}{n}\overset{n}{\underset{i=1}{\text{∑}}}\max{\left\{\text{Sim}(PA_{i},PB_{j})\right\}}_{j=1}^{m}$$

**Figure 5.**
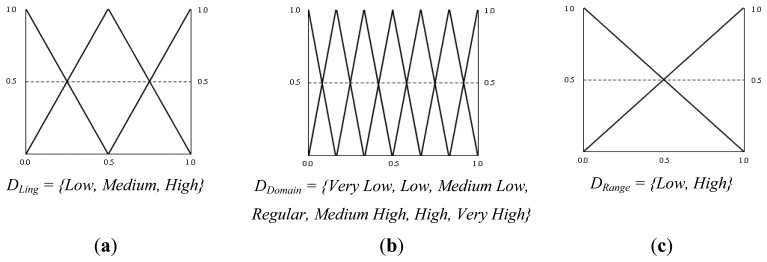
Membership functions and linguistic terms set. (**a**) Linguistic similarity; (**b**) Domain similarity; (**c**) Range similarity.

*Property Similarity*: The similarity between two properties is influenced by three factors: the lexical similarity of their names, the similarity of the classes to which they belong (*Domain*), and the similarity of their types (*Range*). The system used to compute the similarity between the properties of the concepts has those three variables which are calculated in the internal structure module as input, and the output variable represents the similarity between the two properties. Figure 5 shows the membership functions and the linguistic terms set defined for the system variables.

The methods we have used to calculate the domain similarity and the range similarity are the following:

- *Domain Similarity* is the similarity between the classes to which the properties belong. Since in an ontology the same property can belong to more than one class, this is a similar case to the calculation of the hierarchical similarity of parents as shown in Figure 4c.
- *Range Similarity* is the similarity between the types of properties. In the case of object properties, we directly find the similarities between the classes those objects belong to. If these are data properties, the compatibility between the corresponding XML data types must be taken into account. If both are primitive data types, the correspondence between them has to be exact. If at least one type is a derived XML type, their primitive root type must be found and the problem is reduced to comparing two primitive types. This work considers that if two data types are derived from the same primitive type, then they are compatible.

### 4.3. Alignment Layer

The alignment layer is the final layer of the fuzzy rule-based system and its aim is to provide the final similarity index between the concepts taking into account the influence of the number of properties and the value of similarity that properties bring to the final similarity between them. The *Property-wise* similarity is calculated on the internal structure module. Depending on these values, the hierarchical similarity is reinforced or weakened, resulting in a more accurate indicator of the similarity between the concepts. After obtaining the similarities between all concepts and properties of the two ontologies, the alignment module is responsible for selecting the equivalent entities and constructing the final set of alignments. The matching rules are created via the Java API Alignment Format [45], allowing the generation of outputs in different formats.

#### 4.3.1. Confidence Threshold

As the results of applying the fuzzy rule-based system are similarity matrices (real values between 0 and 1), it was necessary to establish a similarity threshold from which entities would be considered equivalent, *i.e.*, the minimum similarity value required to belong to the set of alignments. This confidence threshold can be adjusted according to the characteristics and needs of each application. An optimal confidence threshold should be high enough not to consider equivalent those entities that are not (accuracy), and low enough to get all the correspondences (completeness). In this work the confidence threshold is learned by a genetic algorithm which fitness function is to maximize the F-measure over a set of mapped ontologies. The crossover operator is the standard of two points and the selection mechanism is elitist [46]. The input parameters of the algorithm were: *Population_Size* = 100, *Number_of_Evaluations* = 1,000, *Crossover_Probability* = 0.6, *Mutation_Probability* = 0.1. Finally the threshold which maximizes the F-measure in the analyzed dataset was 0.88.

### 4.4. Fuzzy Inference Engine

The proposed fuzzy rule-based system is a Mamdani type one [47]. This model was chosen because its knowledge base consists of labels and linguistic variables used in human language, which facilitates the population, interpretation and debugging of the rule base. In the design phase of the inference engine we employed the *minimum t-norm* as implication and conjunction operators. For the defuzzification interface we selected the FATI (*First Aggregate, Then Infer*) mode, using the *maximum t-conorm* as the aggregation operator, and the *Largest of the Maxima* (*LOM*) as the defuzzification method [39]. This method consists in selecting the larger of the values that maximize the aggregated membership function as

(5) $$y=\sup\{y|\mu_{B\prime }(y)=\sup\mu_{B\prime }(y)\}$$

where *B'* is the overall fuzzy set obtained after applying the aggregation operator to the initial fuzzy sets and *μ_B_'* is the membership function for *B*'.

### 4.5. Evolutive Learning of the Rule Bases

The rule bases of the fuzzy system were deduced using the genetic algorithm THRIFT [48]. This algorithm is part of the group of methods that follow the Pittsburgh approach [39] and is designed to learn rules of Mamdani type. This method works by using a complete decision table that represents a special case of crisp relation defined over the collections of fuzzy sets. A chromosome is obtained from the decision table by going row-wise and coding each output fuzzy set as an integer. The algorithm uses the rank selection scheme proposed by Baker [49]. The crossover operator is the standard of two points [46], and the fitness function used is the Mean Square Error (*MSE*), so that the best individuals are those that minimize this function. The used dataset has information of 40 ontologies mapped by experts and it was partitioned with a 10-fold Cross Validation method. The input parameters of the algorithm were the following: *Population_Size* = 61, *Number_of_Evaluations* = 1,000, *Crossover_Probability* = 0.6, *Mutation_Probability* = 0.1.

## 5. Experiments and Evaluation

To verify the effectiveness of the proposed method, several experiments were conducted with different ontologies in various domains. We used the tests proposed by the OAEI (*Ontology Alignment Evaluation Initiative*) [50] for evaluating the performance of ontology alignment methods and have compared with other systems. We also conducted several experiments in the domain of sensor networks. From these experiments, we present a general experiment with real sensor network ontologies, and another experiment framed in the forest fire control scenario.

The evaluation measures used in ontology alignment systems are typical of the field of information retrieval. They are *Precision*, *Recall* and *F-Measure*. Given a reference alignment *R* and the resultant alignment *A*, these similarity measures are defined as follows:

- *Precision* describes exactness. It is computed as the fraction of correct instances among those that the algorithm believes to belong to the relevant subset [42]. Given a reference alignment *R*, the precision of some alignment *A* is given by:
  (6) $$\text{Precision}(A,R)=\frac{\left|R\cap A\right|}{\left|A\right|}$$
- *Recall* describes completeness. It is computed as the fraction of correct instances among all instances that actually belong to the relevant subset [42]. Given a reference alignment *R*, the recall of some alignment *A* is given by:
  (7) $$\text{Recall}(A,R)=\frac{\left|R\cap A\right|}{\left|R\right|}$$
- *F-Measure* is used in order to aggregate the result of *Precision* and *Recall* [42]. Given a reference alignment *R* and a number α between 0 and 1, the *F-Measure* of some alignment *A* is given by:
  (8) $$F_{\alpha }(A,R)=\frac{\text{Precision}(A,R)\cdot \text{Recall}(A,R)}{\left(1-\text{α})\cdot \text{Precision}(A,R)+\text{α}\cdot \text{Recall}(A,R\right)}$$

The higher α, the more importance is given to *Precision* with respect to *Recall*. Often, the value α = 0.5 is used. This is the *Harmonic Mean* of *Precision* and *Recall*.

### 5.1. OAEI Tests

For this phase, several experiments with the Ontology Alignment Evaluation Initiative (OAEI) [50] tests datasets were conducted. The results from the Conference and Anatomy tests are presented here, and also a comparison with three of the alignment methods described in Section 3: CODI [30], ASMOV [33], and SOBOM [37]. The experiments were executed in a PC with Intel Core i3 2.0 GHz processor and 4 GB of RAM.

#### 5.1.1. OAEI Conference Test

**Figure 6.**
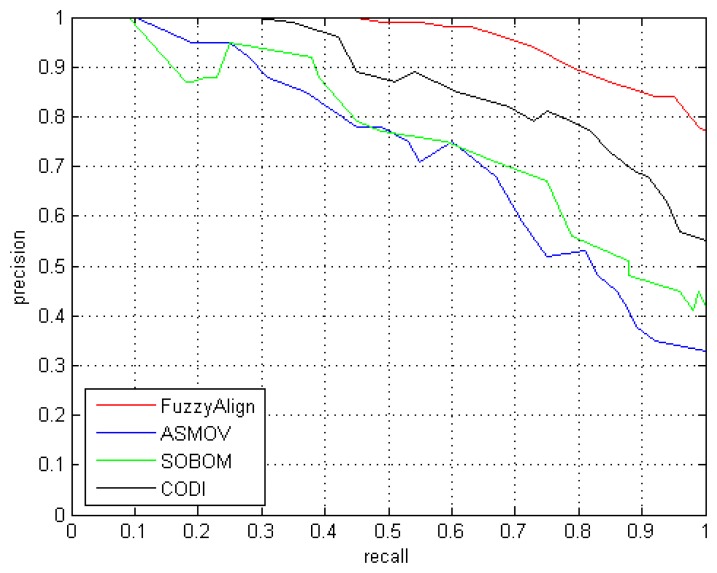
*Precision vs. Recall* curves of the four compared systems in Conference test.

The goal of Conference test [50] is to find all correct matches within a collection of ontologies describing the domain of organizing conferences. Table 2 shows the results of applying the *FuzzyAlign* system with 21 reference alignments, corresponding to the complete alignment space between seven ontologies from the conference data set. These seven ontologies are: *cmt*, *Conference*, *Confof*, *Edas*, *Ekaw*, *Iasted* and *Sigkdd* [50]. Table 3 shows the *Precision*, *Recall*, *F-Measure* and average execution time values obtained by the compared systems, with the same confidence threshold (0.88). In the Table 3 we can see that *FuzzyAlign* system outperformed the other approaches in *Precision*, *Recall* and *F-Measure*. Regarding runtime, the fastest system is ASMOV, and the rest of the systems have a runtime between 2 and 5 min. Figure 6 shows the *precision vs. recall* curves of the 4 methods.

#### 5.1.2. OAEI Anatomy Test

This track consists of two real medical ontologies to be matched [50]. The source ontology describes the Adult Mouse Anatomy (with 2744 classes) while the target ontology is the NCI Thesaurus describing the Human Anatomy (with 3304 classes). In Anatomy test we perform three tasks: *Task #1*, which emphasizes *F-Measure*, *Task #2*, which emphasizes *Precision*, and *Task #3*, which emphasizes *Recall*. The performed tasks use the following configuration parameters:

- *Task #1*. The optimal solution alignment is obtained by using confidence threshold of 0.88.
- *Task #2*. The alignment with optimal *Precision* is obtained by changing the threshold to 0.95.
- *Task #3*. The alignment with optimal *Recall* is generated by changing the threshold to 0.65.

Considering that the ontologies used in this test are very specific to the medical field, the experiment was performed using the UMLS [51] medical databases instead of WordNet as lexical tool. For this reason, we compare only with ASMOV, which is the only approach that uses UMLS to perform the alignment process for the Anatomy test. The results in terms of *Precision*, *Recall* and *F-Measure* are shown in Table 4. As seen in the table, compared to ASMOV, the execution time of *FuzzyAlign* is worse, but the end results are much better. *FuzzyAlign* takes 45 min to process the entire ontologies of the test due to its large amount of information, so it is necessary to improve scalability.

### 5.2. Real Sensor Ontologies Experiment

**Figure 7.**
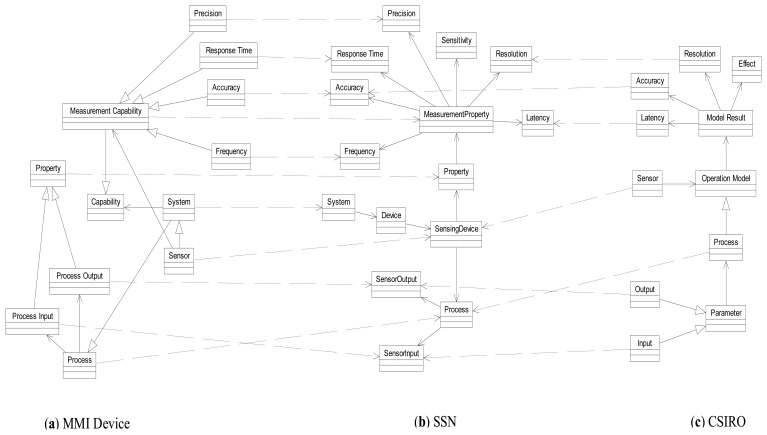
Small fragment of alignments resulting after applying the method to the three selected ontologies. (**a**) MMI Device ontology; (**b**) SSN ontology; (**c**) CSIRO ontology.

This experiment consists on applying the ontology alignment method to three real ontologies belonging to the domain of sensor networks. Due to compatibility issues the chosen ontologies were SSN [25], CSIRO Sensor Ontology [9] and MMI Device Ontology [12]. Initially, the goal is to align the ontologies CSIRO and MMI Device with SSN ontology, because the last one is the most general and comprehensive that has been developed in the domain of sensor networks and it offers alignments with DOLCE ultra lite [26], which is one of the most used global reference ontologies. The alignment method is then applied to align the CSIRO ontology with MMI Device Ontology. In Figure 7 we show a small fragment of the SSN ontology (b) and their links to the MMI Device (a) and CSIRO (c) ontologies. The correspondences found in this fragment are represented by dashed lines.

Table 5 shows the comparative results of applying the four alignment methods to the three selected ontologies in terms of *Precision*, *Recall* and *F-measure*. As can be seen the values of *Precision*, *Recall* and *F-Measure* obtained by *FuzzyAlign* outperform the others, demonstrating the effectiveness of the proposed method in ontologies from sensor networks domain.

### 5.3. Forest Fire Control Scenario

We have performed an experiment for a forest fire control scenario, as mentioned earlier in the introduction. We assume that we have a fixed sensor network spread in a forest, measuring several environmental factors such as humidity, temperature, and presence of smoke and other gases, among others. In this scenario, ontologies play an important role that allows us to organize the vocabulary and the information retrieved from the sensors. With the processing of the values of these indicators and the application of inference rules, fires can be detected early. Once a forest fire alarm has been generated, fire crews go to the affected area.

**Figure 8.**
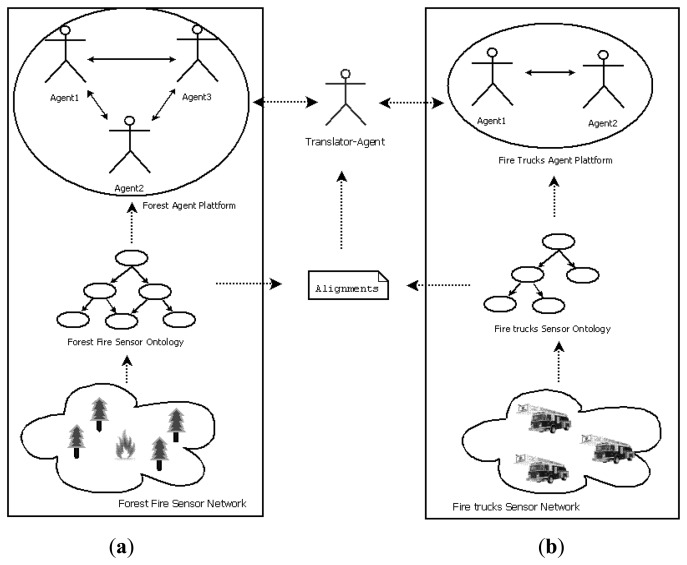
Forest fire control application scenario. (**a**) Forest fire side; (**b**) Fire trucks side.

For the sake of this experiment we will assume there is also a sensor network deployed among the fire trucks, with its own ontology (different from the one used in the forest sensor network). Some of the sensors that can be found in the trucks are position sensors such as GPS, as well as different types of environmental sensors. The key idea is to align both ontologies in order to be able to share information between the sensor networks so that they can take actions that allow rapid control of the fire. One of these actions could be the strategic placement of the fire-fighter teams according to the status of the fire in the different areas. Intelligent control of both networks can be done through agent platforms that are able to proactively take actions in consultation with their own ontologies, which in turn communicates with agents using different ontologies to carry out their tasks. The exchange of messages between agents of different platforms can be performed through a mediating agent, which makes use of the mappings obtained by the ontology alignment algorithm to translate messages from one platform to another. Figure 8 shows a diagram of the proposed application scenario. The overall figure can be analyzed from the bottom up as a multi-level architecture, where sensor networks are at the bottom, on top of them there are the semantics provided by ontologies, and at the top the intelligence layer formed by the agent platforms. We have represented entities in forest environment on the left side (**a**), and the environment of the fire trucks on the right side (**b**).

In this study we have not implemented the agent platform, since we have focused on the process of aligning ontologies. To perform the experiment we have developed two different ontologies with some entities from multiple ontologies known using the Protégé tool [52]. To represent knowledge in the area of sensor networks we have chosen MMI Device ontology [12] and Agent-Based Middleware Approach for Mixed Mode Environments (A3ME) ontology [16]. To represent knowledge related to forest, fire, and everything related to the fire fighters equipment we have used several classes from ontologies of the SWEET suite [23]. The forest fire sensor ontology has 56 classes, and 237 properties, while the fire truck sensor ontology has 78 classes and 342 properties.

**Figure 9.**
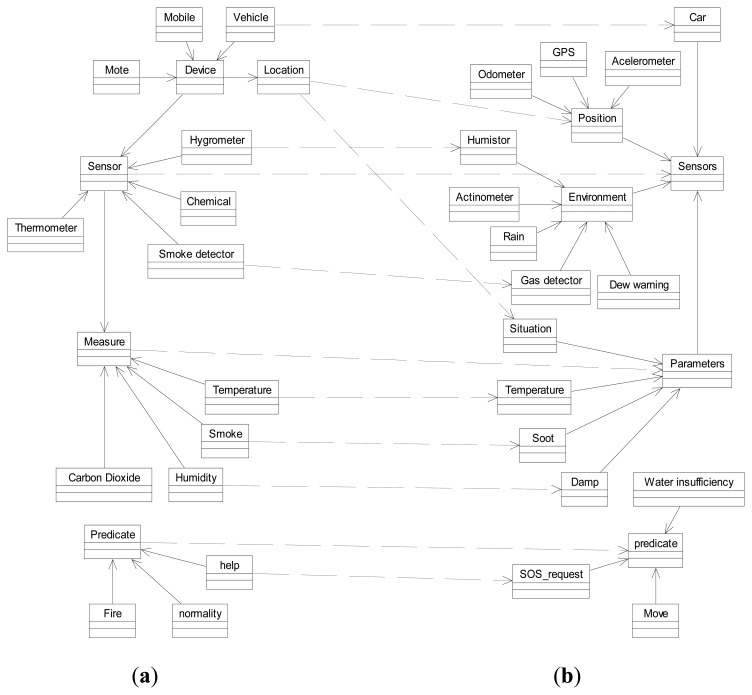
Small fragment of alignments resulting after applying the method to the two ontologies. (**a**) Forest Fire Sensor ontology; (**b**) Fire Trucks Sensor ontology.

Figure 9 shows two small fragments of both sensors ontologies. The correspondences obtained by the ontology alignment algorithm are marked with dashed lines. We have applied *FuzzyAlign* and three of the alignment methods mentioned in the state of the art which have participated in the OAEI competition [50] to these two ontologies to compare the results. These methods were CODI [30], ASMOV [33], and SOBOM [37] and their code and configuration parameters are available online. Table 5 shows the results obtained by the four alignment algorithms with these two ontologies in terms of *Precision*, *Recall*, and *F-Measure*. The confidence threshold used for the selection of the valid alignment was 0.88. This means that we are considering as valid alignment only those mappings whose similarity value is greater than 88%.

Table 6 shows the results of the experiments. We can observe that *FuzzyAlign* achieved the highest average results, outperforming all the other approaches and showing that a high percentage of the obtained matches were correct. Despite this, there were a few mappings undetected because some very specific terms in sensor networks domain were not found in WordNet databases, so it would be desirable to use a specialized sensor thesaurus instead of WordNet.

To illustrate a little better the use of the alignments obtained by the algorithm in the case study of this experiment let's call the Forest Fire Sensor ontology *ontoForest*, and the Fire Trucks Sensor ontology *ontoTrucks*. Suppose that the fire alarm rings and the fire trucks are near the place. The agent in a device that has a temperature sensor in the forest agent platform queries the current temperature value in the ontology and sends a request for assistance through an ACL message to the translator agent. The content of the message is a *help* predicate of *ontoForest*, that has as attributes an object of the class *Temperature* (*float: value*) and an object of the class *Location* (*float: latitude, float: longitude*). The query translator agent uses the mapping table provided by the alignment algorithm and verifies the following matches between classes: *ontoForest.help* = *ontoTrucks.SOS_request*, *ontoForest.Temperature* = *ontoTrucks.Temperature*, *ontoForest.Location* = *ontoTrucks.Situation*, and the following matches between properties: *ontoForest.Temperature.value* = *ontoTrucks.Temperature.val*, *ontoForest.Location.latitude* = *ontoTrucks.Situation.lat*, *ontoForest.Location.longitude* = *ontoTrucks.Situation.long*. Later, the translator agent creates an object from *SOS_request* predicate belonging to *ontoTrucks* ontology in the following way: first it creates an object of class *Temperature* in *ontoTrucks* with the attribute *val* = *ontoForest.Temperature.value* (*Temperature* = *new Temperature* (*val*)), then it creates an object of class *Situation* in *ontoTrucks* with attributes *lat* = *ontoForest.Location.latitude* and *long* = *ontoForest.Location.longitude* (*Situation* = *new Situation* (*lat*, *long*)) and finally it creates the object of class *SOS_request* (*SOS_request* = *new SOS_request* (*Temperature*, *Situation*)). The translator agent will send the message to the agents of the platform of fire trucks, which knowing the location and temperature can decide where to deploy them.

## 6. Conclusions and Future Work

We aim to provide mechanisms to address the problem of semantic heterogeneity caused by the use of different ontologies in the field of sensor networks. In this line, this article describes a Multi-Layer System for the ontology alignment process applied to semantic sensor networks using fuzzy logic techniques. The similarities between entities from different ontologies are computed taking into account semantic and lexical elements and also the relational and internal structures of the ontologies. For linguistic similarity we used the *WordNet* lexical tool, and to compute the semantic similarity we use the *Jaccard coefficient* over contextualized search results of the various entities in sensor subcategory from *DBpedia*. The structural similarity is computed taking into account the relationships of the concepts in the taxonomic hierarchy, as well as elements of its internal structure, such as properties, types, and cardinality.

The system has been tested in domain-specific sensor ontologies and compared to other alignment algorithms in terms of *Precision*, *Recall* and *F-Measure*. The results show that *FuzzyAlign* outperform the other systems using WordNet as lexicon in ontologies with correct lexical constructions. The gain of *FuzzyAlign* could be improved by incorporating more domain concepts that are not in WordNet databases, or by developing a tool that allows the processing of linguistic relationships between specific concepts of sensor networks. It would be desirable to have a specialized thesaurus to handle the lexicon of the specific sensor networks domain terminology. Another problem of using WordNet is that is it only has English language dictionaries, making it impossible to obtain valid alignments between ontologies in other languages. To solve this problem we intend to explore the use of the EuroWordNet tool, which emerged as a project to interconnect several dictionaries of European languages with the same structure as the original Princeton WordNet.

Also we found that the execution time of the system increases when processing too large ontologies, due to the high amount of information, so it is needed to improve the scalability of the framework. In this sense we have thought of using parallel processing techniques that allow us to use multiple processors to concurrently analyze the different parts of the ontologies and thereby optimizing the running time. We are also interested in extending the technique to propose an integration model that allows taking into account the use of other relations between entities in real domains instead of just equivalence. Finally, we intend to do a complete implementation of translation method and the agent platforms in the forest fires environment, with real use cases where we can check the ontology alignment system application in the translation of the messages exchanged by the agents.

## Figures and Tables

**Table 1.** Derivational Linguistic similarity for the concepts *sensor* and *sensing device.*

		**Derivate Words Sets**	**After Stemming**	**Cardinality**
*a*	*sensor*	*A* = {*sensor*, *sense*}	*A* = {*sensor*, *sens*}	|*A*| = 2
*b*	*sensing device*	*B* = {*sensing device*, *sense*}	*B* = {*sens*, *device*}	|*B*| = 2
			*A∩B* = {*sens*}	|*A∩B*| = 1
*Sim*(*a,b*)	0.5			

**Table 2.** Conference test results of *FuzzyAlign* in terms of *F-Measure*.

***F-Measure***	***cmt***	***conference***	***confof***	***edas***	***ekaw***	***iasted***	***sigkdd***
*cmt*		0.87	0.45	0.86	0.88	0.87	0.61
*conference*			0.86	0.88	0.87	0.89	0.80
*confof*				0.59	0.52	0.71	0.82
*edas*					0.44	0.84	0.66
*ekaw*						0.87	0.74
*iasted*							0.77

**Table 3.** Conference test results for the alignment methods in terms of *Precision*, *Recall*, *F-Measure* and *average execution time* in minutes.

***System***	***P***	***R***	***F***	***Average T* (*min*)**
*ASMOV*	0.60	0.41	0.48	0.5
*CODI*	0.86	0.48	0.62	4
*SOBOM*	0.63	0.36	0.46	5
***FuzzyAlign***	**0.90**	**0.60**	**0.72**	**2**

**Table 4.** Anatomy test results for the alignment methods in terms of *Precision*, *Recall*, *F-Measure* and *execution time* (*T*) in minutes.

	***Task #1***				***Task #2***				***Task #3***				***Mean***			
*System*	*P*	*R*	*F*	*T*	*P*	*R*	*F*	*T*	*P*	*R*	*F*	*T*	*P*	*R*	*F*	*T*
*ASMOV*	0.79	0.77	0.78	15	0.86	0.75	0.81	15	0.71	0.79	0.75	15	0.78	0.77	0.79	15
***FuzzyAlign***	**0.97**	**0.91**	**0.94**	**45**	**1.0**	**0.85**	**0.92**	**45**	**0.85**	**0.92**	**0.88**	**45**	**0.94**	**0.89**	**0.91**	**45**

**Table 5.** Results of align the three real sensor network ontologies.

	***MMI Device-SSN***			***CSIRO-SSN***			***MMI Device-CSIRO***			***H-Mean***		
*System*	*P*	*R*	*F*	*P*	*R*	*F*	*P*	*R*	*F*	*P*	*R*	*F*
*ASMOV*	0.84	0.65	0.73	0.72	0.78	0.75	0.79	0.72	0.75	0.78	0.71	0.74
*CODI*	0.78	0.83	0.80	0.81	0.78	0.79	0.86	0.71	0.78	0.82	0.77	0.79
*SOBOM*	0.81	0.74	0.77	0.76	0.81	0.78	0.85	0.63	0.72	0.81	0.72	0.76
***FuzzyAlign***	**0.92**	**0.84**	**0.88**	**0.95**	**0.82**	**0.88**	**0.90**	**0.85**	**0.87**	**0.92**	**0.84**	**0.88**

**Table 6.** Results of applying the four matching algorithms to the two sensor ontologies in terms of Precision (P), Recall (R), and F-Measure (F).

***System***	***P***	***R***	***F***
*ASMOV*	0.82	0.86	0.84
*CODI*	0.90	0.88	0.90
*SOBOM*	0.88	0.82	0.85
***FuzzyAlign***	**0.95**	**0.91**	**0.93**
